# Evaluation of the First Polish Web-Based Intervention Aimed at Improving Cancer Prevention (the PORINA Study)

**DOI:** 10.3390/ijerph15061167

**Published:** 2018-06-04

**Authors:** Maksymilian Gajda, Małgorzata Kowalska, Jan E. Zejda

**Affiliations:** Department of Epidemiology, School of Medicine in Katowice, Medical University of Silesia, 40-055 Katowice, Poland; mkowalska@sum.edu.pl (M.K.); jzejda@sum.edu.pl (J.E.Z.)

**Keywords:** interventional studies, Internet, prevention, cancer, knowledge, web-based

## Abstract

The appropriate level of the society’s health-oriented knowledge is essential for improving the effectiveness of actions to reduce the number of new cases and deaths caused by cancer. The aim of this study was to identify the role of web-based educational campaigns in the field of cancer prevention in Poland. From 14.05.2015 to 13.11.2016 readers of Polish scientific websites were invited to participate in the “PORINA” prospective interventional study. A total of 1118 volunteers (unrepresentative sample) were recruited and randomized (interventional and control groups). After applying the inclusion and exclusion criteria, 463 participants (41.4% of the recruited) qualified for the final analysis; 207 were allocated to the interventional and 256 to the control group. A specially designed internet platform, a self-reported questionnaire (validated during the study) and educational materials which were prepared by a physician specialized in clinical oncology were used. Assessments of participants’ knowledge were based on the authors Cancer Knowledge Index (CKI). The number of subjects with an increase in CKI score was significantly higher for the interventional group with minor changes in the control (*p* < 0.001). The highest increase in CKI scores was obtained in the following demographic groups: females, younger participants, those living in smaller villages and also among the less educated. An overall impact of presented web-based educational intervention was moderate. However, the results obtained confirmed that well-organized intervention supported by oncologists may be useful in cancer prevention.

## 1. Introduction

Some aspects of cancer-related deaths are avoidable primarily due to effective prevention and early diagnosis, as well as treatment and rehabilitation. These factors may reduce the risk of death [1]. From this perspective, the importance of proper level of society’s health-related knowledge cannot be neglected. The perception of the possibility of reducing or eliminating risk factors, the awareness of warning symptoms as well as the need for an early consultation with the physician, and finally the knowledge of preventive examinations are essential for improving the effectiveness of preventive actions [2,3]. Factors such as lack of knowledge, a sense of embarrassment, cultural factors, misconceptions and common myths may hinder prevention campaigns, and generally delay the decision to seek medical advice and thus diagnosis and treatment [3,4,5,6]. It is essential that the Internet is more often used by young people, to whom it is easier to convey appropriate health-related attitudes, including those associated with proper behavior and prevention against cancer. review of current literature revealed that modern research tools based on Internet modalities can be used not only for conducting both questionnaire but also interventional studies [7,8]. Cugelman’s meta-analysis is another important justification for choosing on-line mode of intervention [9].

Unlike many other countries, there are practically no web-based studies conducted in Poland, particularly interventional ones aimed at the assessment of the population’s level of knowledge regarding cancer prevention opportunities [7,8]. On the other hand, neoplastic diseases are a priority in Poland due to the increase in the frequency of their occurrence, as well as the relatively high mortality rate in relation to the situation observed in many of European Union countries [10,11]. It is worth noting that in Poland, as in the USA, this group of diseases are currently the second cause of deaths among the population [6,12]. 

Given the above, it mandatory to identify opportunities, role and usefulness of web-based educational campaigns in the field of cancer prevention in Poland. Therefore, the aim of this study was to assess the impact of the web-based educational intervention on the level of Polish web-users’ cancer knowledge and the possibility of preventing cancer.

## 2. Materials and Methods 

### 2.1. Recruitment Process and IT Solutions

All persons (regardless of their personal cancer history) who were visiting one of the oldest Polish popular science internet portals “Naukowy.pl”, (freely accessible at http://www.naukowy.pl) during the period from 14 March 2015 to 13 November 2016 were invited to participate in the Polish On-line Randomized Intervention aimed at Neoplasm Avoidance (PORINA), a prospective interventional study. According to the study protocol, each visitor, who consented, could participate (open recruitment model) [8]. Subjects’ intent to participate in the study was obtained using an on-line form. After reading the study description, the person interested in participating in the study expressed his consent by providing an email address and selecting clearly described checkbox. Every individual was allowed to have contact with our research team by e-mail in case of any misunderstanding of the principles and course of the PORINA study prior to their declared consent as well as during the study. Participants consent was stored in projects’ database as well as in “cookie files”. This allowed us to control the possibility of re-participation (storage of information about previous visit and declaration regarding participation). Those who did not agree were not asked to participate while the others who agreed to participate were redirected to the author’s electronic research platform. This web-based platform was designed using HyperText Markup Language (HTML), JavaScript, *PHP* language and *MySQL* database. Although we did not collect any personal data (except participants’ e-mail addresses), we used data encryption as well as stored e-mail addresses as separate records. Participants’ e-mail addresses were (with subjects’ agreement) stored in order to inform them about each stage of the study as well as for providing access data to the protected part of PORINA platform. 

A random assignment of subjects to the control or interventional group was performed using a computer algorithm (ratio 1:1). Participants randomized to the intervention group were asked to complete the baseline research questionnaire (phase 1: “F1”). After completing the questionnaire, they were given access to the educational materials prepared by the physician (phase 2: “F2”). A simple quiz was used in order to verify whether they were familiar with the content provided. During the third (and last, “F3”) phase of the study, participants were asked to re-fill the same questionnaire (maintaining 2–6 weeks interval after the end of the first phase) to assess the impact of intervention on their final knowledge, opinions and declarations regarding medical procedures uptake. In contrast, subject from the control group were studied twice using the same questionnaire (at the beginning and the end of the study with the same interval of 2–6 weeks) for its validity, without access to educational materials. 

In order to meet the inclusion criteria it was required to voluntary consent to participate and complete each phase of the study. Participants who did not pass all phases of the study were excluded from the final analysis.

### 2.2. Questionnaire and Educational Materials

The author’s questionnaire survey prepared for the PORINA study was divided into two parts. The first was demographic characteristics (including history of cancer), while the second assessed participants’ knowledge, opinions, and willingness to undergo medical procedures as well as the level of anxiety associated with the diagnosis of cancer. All close-ended questions were written based on well-known published data. The content and choice of questions were consulted with experts in the fields of public health and oncology. The final set was established after a pilot study conducted among small group of people (N = 10, both health professionals and laymen). For research purposes, educational materials were compiled by the author-physician (specializing in clinical oncology). They were shared via the dedicated study’s web platform in the second phase of the study. Participant could watch an 11-min multimedia presentation featuring animations with commentary by the physician or (e.g., in case of technical difficulties) a basic version with text and images. They were given information on major determinants of cancer, relationship between lifestyle and risk of disease, preventive measures, and the importance of recognizing alarm symptoms and the actual recommendations of “European Code Against Cancer” [13]. The full version of the questionnaire and educational materials (in Polish) are available from the corresponding author on request.

### 2.3. Data Analysis

Participants’ knowledge was evaluated based on the author’s questionnaire and value of the original Cancer Knowledge Index (CKI) which was derived from answers to selected 20 questions and expressed as discrete quantitative variables. All questions were equivalent, with one point for correct and zero point for an incorrect answer. CKI was calculated as the sum of the points obtained for the answer to each question, with the minimum possible value of 0 and maximum of 20 points. For the purposes of the analysis, the following evaluation criteria were adopted: “low CKI” included values <1 tertile, “average CKI” comprised values of the closed interval between the first and second tertile while the values above the second tertile denoted “high CKI”. 

Data were analysed with R software capabilities [14], with the elements of descriptive as well as analytical statistics. statistical significance was set at *p* < 0.05. For the interpretation of the differentiation of quantitative variables in independent groups defined e.g., by selected demographic factors, non-parametric U-Mann-Whitney or ANOVA Kruskal-Wallis tests were used, while chi^2^ test was used for qualitative variables. In order to assess the reliability of the CKI scale, the Cronbach’s α statistic was calculated for which the values above 0.7 were considered reliable [15]. In addition, Cohen Kappa statistics with 95% confidence intervals (CI) was calculated to assess the consistency of responses provided by the surveyed from control group (preliminary versus final questionnaire). The criteria proposed by Landis and Koch were used for interpretation [16]. Since paired variables were compared to evaluate the impact of educational intervention, each time during the statistical analysis appropriate tests were used for such type of variable. Wilcoxon pair test was used for qualitative and suitable for ranging paired variables. The McNemar statistical test was used for qualitative variables expressed on a dichotomous scale. Cliff δ statistics were calculated for the evaluation of the intervention effect size. It was assumed that the value of δ > 0.474 means a large effect size of the intervention, the value of 0.33–0.474 average, the value of 0.147–0.33 denote low, while the value of δ < 0.147 indicates a marginal and negligible effect [17].

### 2.4. Ethical Aspects

Data collection and storage were carried out in accordance with applicable laws and the approval of the Bioethical Committee of the Medical University of Silesia in Katowice, Poland (KNW/0022/KB1/146/14). Detailed description of the proceedings can be provided on request.

## 3. Results

### 3.1. Basic Characteristics of the Study Population

**Figure 1:**
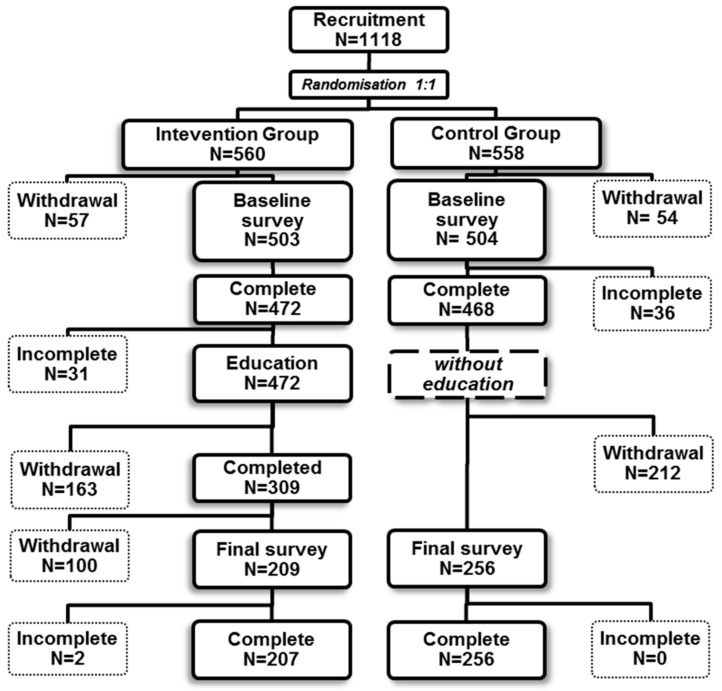
Summary of the number of participants in each study phase (including dropouts).

A total of 1118 volunteers were recruited and randomly assigned to the control (N = 558) or interventional group (N = 560). After considering the inclusion and exclusion criteria (subjects’ agreement and questionnaire completeness), 463 participants were included in the final analysis (41.4% of recruited); 207 subjects with intervention (37%) and 256 (45.9%) for the control group. Both study groups did not differ significantly in terms of selected demographic variables, regarding family nor individual burden of cancer (Table 1). Figure 1 illustrates a detailed study scheme taking into account the number of participants in each phase of the study.

### 3.2. The Validity and Reliability of the Study Instrument

Numbers and percentages of responses to selected questions (comparing results from preliminary and final questionnaire) separately for the intervention and control group are shown in Table 2. The satisfactory repeatability was obtained for the whole questionnaire. The Cohen Kappa statistics values ranged from 0.86 (95% CI: 0.83–0.88) to 0.77 (95% CI: 0.74–0.81). Raw value of Cronbach’s α statistics for the CKI scale was 0.58 (95% CI 0.53–0.63), while the standardized value was 0.59. Moreover, the presence of floor and ceiling effects could be excluded as the percentage of individuals with the lowest and highest CKI scores did not exceed 0.2%. 

### 3.3. Findings from the Intervention

A majority of subjects (78.4%) considered their cancer-related knowledge as insufficient, with more than 92% stating a desire to improve it. The baseline level of participants’ cancer-related knowledge measured by the correctness of the answers to the questions varied. The median value of CKI for all participants was slightly lower in the baseline survey compared to that calculated for the final survey (14 and 16, respectively). There was no significant difference between the control and intervention group in relation to CKI value (*p* = 0.2), while the median of CKI absolute change (final minus baseline CKI) differed statistically significantly (*p* < 0.001). Detailed results are presented in Table 3. 

**Figure 2:**
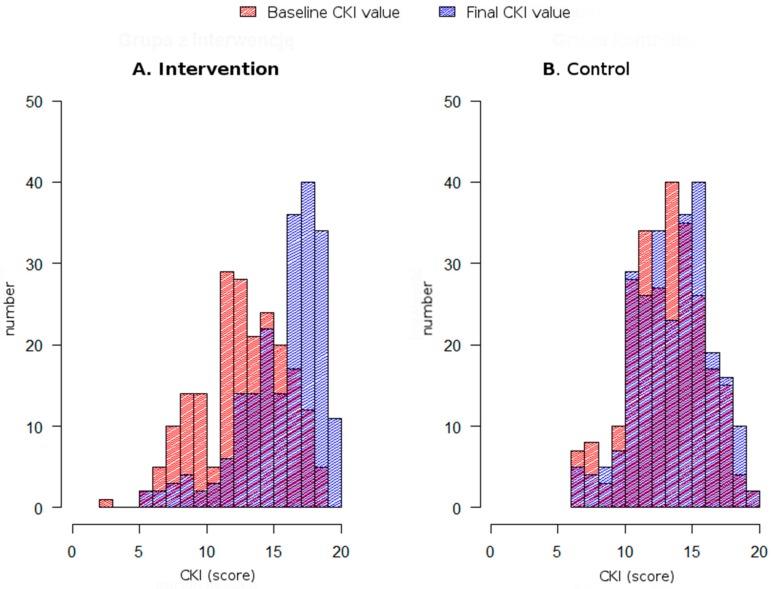
CKI scores before (grey) and after (black) educational intervention in the interventional (**A**) and control (**B**) group.

After educational intervention, an increase of the average CKI value was obtained (from 13.1 to 15.9 points, *p* < 0.001), without any significant changes for the control group. In addition, Figure 2 shows distributions of CKI (baseline and final values) in the intervention and control group. The higher increase in CKI values was found in participants with lower education, women, younger subjects (under 41 years old) and living in rural areas as well as small towns (≤100,000 people). The non-medical participants and those wishing to improve their knowledge also benefited more from the intervention. A statistically significant increase of 20% in the CKI values was found in subjects without oncological history (family and individual). The Cliff δ values obtained in the study confirmed the high effect of the intervention with the exception of subjects with higher medical education, for which the effect was moderate (δ = 0.35).

## 4. Discussion

The development of information technology (IT) and the increase in the number of people with access to the Internet is a viable medium frequently used for providing health-related contents. Many publications have demonstrated the effectiveness of interventions aimed at improving health behaviors. Educational campaigns encourage people to participate in preventive examinations [18,19] and to search for additional information [20,21]. New opportunities to educate patients may be useful for health care providers and insurers [19] as well as for various groups of health professionals, such as nurses [20,22] or physicians [23]. It was also considered appropriate to conduct web-based educational programs addressed to patients with a diagnosis of cancer, including a growing number of “cancer survivors” in order to reduce disparities in access to information and examinations due to the patients’ low economic status [24,25,26,27]. The results of Cugelman et al. demonstrated the effectiveness of actions aimed at the behavioral change [9]. Moreover, the possibility of reaching out to a wider audience at reasonable costs was observed. This is particularly important for modification of young people’s behaviors, which include the frequent use of new ways of communication such as social media [28]. In general, three groups of interventional studies have been identified [29]. The first group includes enriched informational and environmental methods aimed at providing access (using hyperlinks) to educational materials or guidelines of scientific societies. The second group includes information exchange (via chat rooms or internet discussion boards). The last group covers supplementary methods, such as providing educational content with short message service (SMS) or e-mail [29].

A review of available published data indicates that our study is the first of its kind in Poland. For these reasons, we hypothesized that educational interventions conducted remotely might have the potential to enhance the level of knowledge in Polish conditions. Due to the lack of existing Polish IT solutions that would meet the requirements of the study protocol (including the need for randomisation), we decided to develop subject-dedicated IT platform. Similarly, authors of other studies also used their own IT solutions [19,30] or did not disclose information about platform used [31]. 

The recruitment of the participants to our study was based on the “open model of recruitment” which is likely to be affected by selection or volunteer bias [31,32,33,34]. As a result, the generalization of the results may be impossible [35]. Rosenthal and Rosnow revealed that volunteers are better educated, wealthier, healthier, more empathic and intelligent [36]. It has also been confirmed that women and people without addictions are more likely to voluntarily participate in the study [37]. The profile of PORINA study’s participants is consistent with the above observations, especially with regard to the level of education and feminization. However, a recruitment of compliance with privacy and data protection policies, and finally ethical considerations, excluded the possibility of determining whether study participants differed significantly from non-responders. Another important point of discussion is that the questionnaire included close-ended questions. This type of question may overstate, while open-ended may understate the actual knowledge. However, there is no certainty regarding which form better reflects the cancer-related knowledge [38]. It is also worth emphasizing that our study population was intentionally made up of people mostly with negative personal history of malignant neoplasms, as the most appropriate recipients of educational campaigns aimed at primary and secondary prevention.

Social cognitive theory (SCT), theory of reasoned action/planned behavior (TPB) and transtheoretical model (TTM) are the most often used theoretical models for constructing behavioral interventions [29]. Our intervention was based on this last model, as in order to assess the individual’s readiness to change behavior. It seems that participation in the study itself can become a motivation for self-exploration of the subject matter. Lana et al. found that even the completion of the questionnaire may have an educational aspect [31]. This is consistent with the results of our study, which also demonstrated within the control group there was a significant increase in the number of correct answers to three questions. There has been a slight increase in the number of participants who denied the truth of the statement that “*tobacco smokers with lung cancer can only blame themselves*”. This may be related to a more liberal, empathic approach to the responsibility (blame) for smokers who develop the disease. Such interpretation applies to both control and intervention groups. Such a change of attitudes therefore should be interpreted positively, especially in the context of social stigmatization of patients with the diagnosis of cancer [39]. It should also be noted that also other authors observed surprising and sometimes difficult to interpret results of educational intervention [40]. 

Despite the moderate reliability of the CKI scale (Cronbach’s α of 0.59), the questionnaire validation was successful, as we reached very good repeatability (assessed using Cohen’s Kappa). These results allow us to draw a legitimate conclusions based on a developed research tool. The “pretest-posttest” model is commonly used to evaluate the effect of an intervention programs [8,22,41,42,43]. Statistical tests such as Cohen d or Hedges g (parametric) or Cliff δ (non-parametric) can be used to evaluate the effect size of intervention [17,44]. The PORINA study’s results show a statistically significant increase in the values of CKI (by 18%) in the group of people who underwent the educational intervention. It is worth mentioning that the largest change was among people with the lowest level of education (CKI increase of 25%). Cliff’s δ test results confirm the significant effect of educational intervention (Table 3). Due to the aforementioned lack of this type of research in Poland, the results were referred to the experiences of other authors dealing with the assessment of the effectiveness of various interventions in other countries. Table 4 contains a set of data that disclose the effects of cited interventional studies in which final result was expressed by relative differences between endpoints (after intervention) and initial values (before intervention). It is noteworthy that similar to our results, Robb et al. [43] found that the awareness of risk factors for colorectal cancer was significantly higher (comparing to the control group) among participants who underwent educational intervention. Lana et al. demonstrated an opportunity to reduce the risk of lifestyle-related cancers by affecting the change in anti-health behavior. In the case of the group educated only via Internet, reduction in the authors’ Total Cancer Behavioral Risk (TCBR) was estimated at 16.9%, while in the short message service (SMS) group, the severity of the risky behavior was reduced by 27% [31]. Our study showed similar results. 

The lack of participants’ interest in the study’s subject or in improvement of their knowledge are potential limitations of the effectiveness of web-based interventions. Another important reason is dropout of subjects during the project [31,45]. These factors lead to a decrease in the response ratio [32,46] and selection bias. In our study, the dropout rate (the ratio of the number of people who completed the study to all enrolled) was 58.6%. This is consistent with other authors report [45]. The highest percentage of dropout participants was registered among those who were younger, less educated and inhabiting less urbanized areas. Also in the study by Bantum et al. less educated participants resigned more frequently [24]. A more detailed understanding of the determinants of “dropout” phenomenon seems to be crucial for improving retention rates of participants. Possible solutions to this problem include the use of individual invitations, a refined research questionnaire, reminders, and finally both cash and non-monetary incentives [7,8,18,22,37,47]. During our study, only reminding messages were used, however we did not record the number of messages sent and we are not able to assess their impact.

Among possible reasons for a relatively poor effect of the intervention, one can suspect the use of inappropriate tools, incomprehensible or inappropriate form of educational materials, or the effect of extensive expectations such as the desire to change many health behaviors simultaneously [8,31]. This phenomenon cannot be ruled out in the case our intervention due to the multifaceted content of the educational material used. Perhaps narrowing the subject (e.g., to only specific type of cancer) would allow to achieve a higher participants’ focus on the subject and, as a result, would improve the effectiveness of the intervention itself. According to many authors, the effectiveness of interventions could also be improved with the individualization of educational content [21,28,40,41], feedback [46] and promoting the use of Internet among the elderly [7]. It is also worth considering the possibility of simultaneous use of more than one form of communication during the intervention, e.g., both the telephone and web-based versions [31,47]. Increased control of the study participant’s visit to the web platform leads to increased page views and increases time spent browsing the internet platform. It was also demonstrated that larger number of completed educational modules has a positive impact on the level of knowledge gained [48].

Given the above, it cannot be excluded that the actual level of cancer-related knowledge of the Polish society is even lower than that shown by the results of our study. One could expect a better effect of educational intervention under condition of increasing participation less educated people, who as shown by our study, resigned more often while at the same time gained the greatest benefit from the intervention. Unmistakable advantage of web-based interventions, unlike traditional methods, is the possibility to check the completeness of the answers given and to inform the participants about the need to provide requested information [35]. This solution minimizes the risk of leaving questionnaires unanswered while providing immediate access to the saved information and the possibility to correct any errors [35,49]. Application of the IT platform for the construction and presentation of electronic questionnaires give an opportunity to use previously unavailable, interactive solutions, such as providing additional explanatory information or even multimedia content [19,35,49]. 

The presented study is an example of implementation of the above solutions, although no separate assessment of their correctness nor implementation was planned. Moreover, our results may have general interest for the readers who play significant role in public health and focus on health promotion not only in study region, but also in other countries dealing with this unsolved problem.

## 5. Conclusions

The overall impact of the presented web-based educational intervention was moderate. However, its effectiveness in improving cancer-related knowledge was proved in some subgroups, especially in those with the lowest level of education. It should be assumed that well organized web-based intervention may also be useful in cancer prevention in Poland. Before the implementation of such web-based intervention would be possible on a wider scale, additional stages of research are undoubtedly necessary. Therefore, new and larger prospective trials (with the traditional way of information delivery as a comparison with web-based intervention) should assess the utility of well-validated web-based learning tools in improving health behavior taking positive cues from our preliminary results.

## Figures and Tables

**Table 1:** Characteristics of subjects in the interventional and control group.

Characteristic		Group			
	Variables	Overall	Control	Interventional	*p*
		N = 463	N = 256	N = 207	
**Age (years)**	median (IQR)	33 (22–47)	31 (22–47)	35 (21–47)	0.9 ^#^
**Baseline CKI**	median (IQR)	14 (12–16)	14 (12–16)	14 (11–16)	0.2 ^#^
**Final CKI**	median (IQR)	16 (13–18)	14 (12–16)	17 (15–18)	<0.001 ^#^
**Gender**	Male	179 (38.7%)	100 (39.1%)	79 (38.2%)	0.8 ^&^
	Female	284 (61.3%)	156 (60.9%)	128 (61.8%)	
**Place of residence**	Village	101 (21.8%)	60 (23.4%)	41 (19.8%)	0.6 ^&^
	Small city	117 (25.3%)	64 (25.0%)	53 (25.6%)	
	Large city	245 (52.9%)	132 (51.6%)	113 (54.6%)	
**Level of education**	Primary	29 (6.3%)	12 (4.7%)	17 (8.2%)	0.2 ^&^
	Secondary	167 (36.1%)	93 (36.3%)	74 (35.7%)	
	High school	238 (51.4%)	131 (51.2%)	107 (51.7%)	
	Higher medical	29 (6.3%)	20 (7.8%)	9 (4.3%)	
**Medical occupation**	No	388 (83.8%)	215 (84.0%)	173 (83.6%)	0.9 ^&^
	Yes	75 (16.2%)	41 (16.0%)	34 (16.4%)	
**Positive family history of cancer**	*-overall*				
	No	120 (25.9%)	61 (23.8%)	59 (28.5%)	0.3 ^&^
	Yes	343 (74.1%)	195 (76.2%)	148 (71.5%)	
	*-parents*				
	No	333 (71.9%)	180 (70.3%)	153 (73.9%)	0.4 ^&^
	Yes	130 (28.1%)	76 (29.7%)	54 (26.1%)	
	*-grandparents*				
	No	250 (54.0%)	141 (55.1%)	109 (52.7%)	0.6 ^&^
	Yes	213 (46.0%)	115 (44.9%)	98 (47.3%)	
	*-siblings*				
	No	440 (95.0%)	241 (94.1%)	199 (96.1%)	0.4 ^&^
	Yes	23 (5.0%)	15 (5.9%)	8 (3.9%)	
**Participant with diagnosis of cancer**	No	413 (89.2%)	225 (87.9%)	188 (90.8%)	0.4 ^&^
	Yes	50 (10.8%)	31 (12.1%)	19 (9.2%)	
**Participant treated oncologically**	No	415 (89.6%)	229 (89.5%)	186 (89.9%)	1 ^&^
	Yes	48 (10.4%)	27 (10.5%)	21 (10.1%)	
**Self-declaration of sufficient level cancer-related level of knowledge**	No	362 (78.2%)	201 (78.5%)	161 (77.8%)	0.9 ^&^
	Yes	101 (21.8%)	55 (21.5%)	46 (22.2%)	
**Self-declaration of willingness to improve the level of cancer-related knowledge**	No	35 (7.6%)	20 (7.8%)	15 (7.2%)	0.9 ^&^
	Yes	428 (92.4%)	236 (92.2%)	192 (92.8%)	

CKI—Cancer Knowledge Index; IQR—interquartile range; *p*—significance of the U Mann-Whitney test for quantitative variables (^#^) and χ^2^ test for qualitative variables (^&^); Small city—with ≤100,000 inhabitants; Large city—with >100,000 inhabitants.

**Table 2:** Impact of the educational intervention to answer of particular questions in interventional and control group.

	Group with Education Interventional				Group without Education Control			
Statement (Percentage of Agreement)	B	F	R	*p*	B	F	R	*p*
Cancer is a destiny which cannot be prevented	86.0	90.3	4.3	0.1	84.8	85.2	0.4	0.99
Consuming smaller amounts of food slows down while larger ones accelerates the growth of cancer	58.0	70.0	12.0	0.002	61.7	59.0	−2.7	0.4
Patients treated with chemotherapy should drink red beet juice	9.2	50.7	41.5	<0.001	10.2	12.9	2.7	0.3
Tobacco smokers with lung cancer can only blame themselves	53.1	49.8	−3.3	0.4	55.5	46.9	−8.6	0.009
Cancer is always pain and suffering	40.1	75.8	35.7	<0.001	42.6	50.8	8.2	0.009
A person diagnosed with cancer can work	78.3	87.9	9.6	0.002	81.2	82.4	1.2	0.7
It’s better not to remove suspicious skin lesions as they will become malignant	66.7	76.3	9.6	0.006	73.0	75.0	2.0	0.5
Cancer is contagious	93.7	94.7	1.0	0.8	95.3	94.9	−0.4	0.99
It is better not to perform a biopsy of the cancer, because the disease will spread throughout the organism	75.8	87.4	11.6	<0.001	85.2	85.2	0.0	0.99
There is no treatment in the hospice	68.6	83.6	15.0	<0.001	70.7	69.9	−0.8	0.9
The vaccine against HPV human papilloma virus may protect against cervical cancer	55.1	70.0	14.9	<0.001	57.0	60.9	3.9	0.2
Only women get breast cancer	67.6	95.2	27.6	<0.001	70.7	76.6	5.9	0.01
Lung cancer occurs only in smokers	98.6	98.1	-0.5	0.99	97.7	97.7	0.0	0.99
Smokers have a higher risk of developing pancreatic cancer	48.8	72.9	24.1	<0.001	50.8	55.9	5.1	0.1
Blacks do not get melanoma skin cancer	45.9	76.8	30.9	<0.001	51.6	51.6	0.0	0.99
Breast cancer occurs predominantly in women under the age of 30, rarely in later life	87.4	87.0	−0.4	0.99	84.0	85.5	1.5	0.6
The presence of blood in stool can be a symptom of cancer of the colon cancer	88.9	96.1	7.2	0.005	90.2	91.8	1.6	0.6
Invalid lifestyle may increase the risk of cancer	97.6	97.1	-0.5	0.99	96.1	96.9	0.8	0.7
It is enough to apply sunscreen once a day preferably in the morning to protect against ultraviolet UV	84.5	87.0	2.5	0.4	84.4	87.5	3.1	0.2
Normal level of tumor markers excludes the diagnosis of cancer	50.2	84.1	33.9	<0.001	52.7	57.4	4.7	0.1

Legend: B—baseline level of agreement (first survey); F—final level of agreement (second survey); R—relative difference presented as percentage; *p*—significance in McNemar’s test.

**Table 3:** Change in CKI scores in the control and intervention group with the effect sizes.

		Group with Education (Interventional)					Group without Education (Control)				
		B	F	D	*p*	δ	B	F	D	*p*	δ
***Overall***		14	17	18	<0.001	0.53	14	14	0	<0.001	0.09
***By category***											
Age group (years)	<24	13	17	18	<0.001	0.55	14	14	0	0.3	0.05
	24–41	14	16	18	<0.001	0.48	14	15	0	0.004	0.12
	>41	13	17	20	<0.001	0.56	14	14	5	0.01	0.1
Gender	Male	14	17	18	<0.001	0.50	14	14	0	0.02	0.09
	Female	13	17	20	<0.001	0.54	14	14	0	0.002	0.09
Number of inhabitants in the place of residence	≤100,000	13	17	18	<0.001	0.50	14	14	0	0.002	0.1
	>100,000	13	17	20	<0.001	0.55	14	15	0	0.02	0.08
Level of education	Primary	12	17	25	0.002	0.64	14	14.5	−6	0.5	0.01
	Secondary	13	17	15	<0.001	0.42	13	13	0	0.02	0.09
	High school	13	17	21	<0.001	0.62	14	14	0	0.005	0.09
	Higher medical	17	18	5	0.2	0.35	16	16	5	0.03	0.2
Medical occupation	No	13	17	20	<0.001	0.55	14	14	0	<0.001	0.09
	Yes	16	17	11	<0.001	0.40	15	16	0	0.09	0.1
Positive family history of cancer	No	12	16	20	<0.001	0.49	13	13	0	0.2	0.04
	Yes	14	17	18	<0.001	0.56	14	15	0	<0.001	0.11
Participants with diagnosis of cancer	No	13	17	20	<0.001	0.52	14	14	0	<0.001	0.09
	Yes	15	18	18	<0.001	0.67	14	15	5	0.1	0.14
Treated oncologically	No	13	17	20	<0.001	0.54	14	14	0	<0.001	0.09
	Yes	15	17	18	0.005	0.42	14	14	0	0.2	0.08
Self-esteem level of knowledge about cancer as sufficient	No	13	17	20	<0.001	0.56	13	14	5	<0.001	0.12
	Yes	14.5	17	14	<0.001	0.44	15	15	0	0.6	0.02
The readiness to improve the level of cancer-related knowledge	No	15	18	20	0.004	0.59	14.5	14	0	0.5	0.01
	Yes	13	17	18	<0.001	0.53	14	14	0	<0.001	0.1

Legend: B—baseline median value of CKI (first survey); F—final median value of CKI (second survey); D—relative difference between final and baseline values of CKI (in %); *p*—Wilcoxon’s significance level for paired variables; δ—Cliff’s delta; a measure of effect size.

**Table 4:** Comparison of the effectiveness of selected web-based educational interventions.

What Was the Purpose of the Intervention?	The Size of the Study Group	Effect of Intervention (Relative Difference in Percentages) ^1^	Author and Year of Publication
Reduce the lifestyle-related risk of cancer		Intervention	Lana, 2014 [31]
	177	standard: 16.9%	
	244	supplemented with SMS: 27.2%	
Cancer-related knowledge of nurses	48	18.3%	Choma, 2015 [22]
Insomnia level	156	−4.3%	Bantum, 2014 [24]
Strenuous exercise	156	37.2%	
Stretching	156	32.6%	
The level of awareness of medical staff	29	12.1%	Park, 2014 [30]

^1^ Calculated with the equation: $\frac{\left(\mathrm{final}\;\mathrm{value}\;-\mathrm{baseline}\;\mathrm{value}\right)\;*\;100\%}{\mathrm{baseline}\;\mathrm{value}}.$
